# Interleukin-6 Elevation Is a Key Pathogenic Factor Underlying COVID-19-Associated Heart Rate-Corrected QT Interval Prolongation

**DOI:** 10.3389/fcvm.2022.893681

**Published:** 2022-05-19

**Authors:** Pietro Enea Lazzerini, Riccardo Accioli, Maurizio Acampa, Wen-Hui Zhang, Decoroso Verrengia, Alessandra Cartocci, Maria Romana Bacarelli, Xiaofeng Xin, Viola Salvini, Ke-Su Chen, Fabio Salvadori, Antonio D’errico, Stefania Bisogno, Gabriele Cevenini, Tommaso Marzotti, Matteo Capecchi, Franco Laghi-Pasini, Long Chen, Pier Leopoldo Capecchi, Mohamed Boutjdir

**Affiliations:** ^1^Department of Medical Sciences, Surgery and Neurosciences, University of Siena, Siena, Italy; ^2^Stroke Unit, University Hospital of Siena, Siena, Italy; ^3^National Standard Laboratory of Pharmacology for Chinese Materia Medica, School of Pharmacy, Nanjing University of Chinese Medicine, Nanjing, China; ^4^Department of Pharmacy, Maanshan People’s Hospital, Maanshan, China; ^5^Department of Medical Biotechnologies, University of Siena, Siena, Italy; ^6^Department of Respiration, Affiliated Jinling Hospital School of Medicine, Nanjing University, Nanjing, China; ^7^School of Medicine, Nanjing University, Nanjing, China; ^8^VA New York Harbor Healthcare System, New York, NY, United States; ^9^SUNY Downstate Health Sciences University, New York, NY, United States; ^10^NYU School of Medicine, New York, NY, United States

**Keywords:** QTc interval, systemic inflammation, interleukin-6, guinea pig model, ventricular electrical remodelling, action potential duration, I_Kr_ current, COVID-19

## Abstract

**Background:**

Heart rate-corrected QT interval (QTc) prolongation is prevalent in patients with severe coronavirus disease 2019 (COVID-19) and is associated with poor outcomes. Recent evidence suggests that the exaggerated host immune-inflammatory response characterizing the disease, specifically interleukin-6 (IL-6) increase, may have an important role, possibly *via* direct effects on cardiac electrophysiology. The aim of this study was to dissect the short-term discrete impact of IL-6 elevation on QTc in patients with severe COVID-19 infection and explore the underlying mechanisms.

**Methods:**

We investigated the following mechanisms: (1) the QTc duration in patients with COVID-19 during the active phase and recovery, and its association with C-reactive protein (CRP) and IL-6 levels; (2) the acute impact of IL-6 administration on QTc in an *in vivo* guinea pig model; and (3) the electrophysiological effects of IL-6 on ventricular myocytes *in vitro*.

**Results:**

In patients with active severe COVID-19 and elevated IL-6 levels, regardless of acute myocardial injury/strain and concomitant QT-prolonging risk factors, QTc was significantly prolonged and rapidly normalized in correlation with IL-6 decrease. The direct administration of IL-6 in an *in vivo* guinea pig model acutely prolongs QTc duration. Moreover, ventricular myocytes incubated *in vitro* with IL-6 show evident prolongation in the action potential, along with significant inhibition in the rapid delayed rectifier potassium current (I_Kr_).

**Conclusion:**

For the first time, we demonstrated that in severe COVID-19, systemic inflammatory activation can *per se* promote QTc prolongation *via* IL-6 elevation, leading to ventricular electric remodeling. Despite being transitory, such modifications may significantly contribute to arrhythmic events and associated poor outcomes in COVID-19. These findings provide a further rationale for current anti-inflammatory treatments for COVID-19, including IL-6-targeted therapies.

## Introduction

It is well accepted that heart rate-corrected QT interval (QTc) on the surface electrocardiogram (ECG) reflects the duration of the action potential (AP) in ventricles, in turn as a result of the sequential activation of inward depolarizing currents, mostly through sodium and calcium channels, and outward repolarizing currents, principally through potassium channels (1, 2). Whenever a dysfunction of one or more of these channels results in an inward shift in the overall balance of currents, the AP duration (APD) prolongs and, therefore, the QTc (1, 2). A particularly critical role is played by the ether-a-go-go-related gene potassium (K^+^) channel (hERG), conducting the rapid delayed rectifier current (I_Kr_), whose blockade underlies most cases of drug-induced QTc prolongation and beyond (2, 3).

Prolongation of QTc is a recognized risk factor for life-threatening ventricular arrhythmias (VAs) and sudden cardiac death (2). Large studies and meta-analyses provided evidence that both QTc prolongation and malignant VAs are prevalent in patients with severe coronavirus disease 2019 (COVID-19) (4–9) and are associated with poor outcomes, comprising severe illness, intensive care unit (ICU) admission, and mortality (7, 9, 10). Several factors probably contribute to COVID-19-associated QTc prolongation, and the most investigated are repurposed pharmacological treatments that block the hERG-K^+^ channel, such as antimalarials, macrolides, and protease inhibitors, and acute myocardial injury/strain, possibly due to direct virus invasion and/or hypoxia-induced cell damage or stretch (11). Moreover, severely ill patients with COVID-19 frequently present with other concomitant QT-prolonging risk factors, that is, pre-existing cardiac diseases, electrolyte disturbances, and medications employed in the critical patient (proton pump inhibitors, antiemetics, and anesthetics) (11).

Nevertheless, increasing evidence suggests that the exaggerated host immune-inflammatory response characterizing the disease, not infrequently a true cytokine storm, may have an even more important role in the phenomenon, since this condition is invariably present in all patients with severe COVID-19 (11). Several authors reported that in patients with COVID-19, QTc prolongation and related arrhythmic events can develop even in the absence of (or after adjustment for) the above-mentioned risk factors/alterations, and conversely associated with disease severity and degree of systemic inflammation (5, 6, 12–15). Indeed, accumulating data demonstrated that systemic inflammatory activation due to acute non-COVID-19 infections or other inflammatory diseases can directly prolong QTc, *via* cytokine-mediated electrophysiological effects on the heart (16–18). Interleukin-6 (IL-6), which is commonly and significantly elevated in severe COVID-19 (11), seems to play a major role by modulating ion channel expression and functions in ventricular myocytes (17, 19). Accordingly, a retrospective study by Gulletta et al. (20) found that QTc duration correlated with the C-reactive protein (CRP) levels in patients hospitalized with COVID-19. Moreover, another retrospective analysis on a subset of a large cohort of COVID-19 patients preliminarily suggests an association between IL-6 levels and their QTc maximum measurements over at least 2 days (6).

The present study focused on dissecting the short-term discrete effects of systemic inflammatory activation, specifically IL-6 elevation, on QTc in patients with severe COVID-19 and exploring the underlying basic mechanisms. Thus, we investigated: (1) the QTc duration in patients with COVID-19 during the active phase and recovery, and its association with CRP and IL-6 levels; (2) the acute impact of IL-6 administration on QTc in an *in vivo* guinea pig model; and (3) the electrophysiological effects of IL-6 on ventricular myocytes *in vitro*.

## Patients and Methods

The data underlying this study are available in the article and its online Supplementary Material.

The local ethical committee (Comitato Etico Regionale per la Sperimentazione Clinica della Regione Toscana, Sezione Area Vasta Sud Est) approved the study, and patients from all groups gave their oral and written informed consent in accordance with the principles of the Declaration of Helsinki. All experiments involving animals were approved by the local Animal Care and Use Committee.

### Study Populations

The impact of IL-6 on QTc in COVID-19 was evaluated by prospectively enrolling 33 consecutive patients admitted to our University Hospital for active severe disease. COVID-19 diagnosis was based on the presence of (1) symptoms suggestive of COVID-19 illness and (2) positive clinical polymerase chain reaction assays for severe acute respiratory syndrome coronavirus-2 (SARS-CoV-2) on the nasopharyngeal swab. Severe COVID-19 infection was defined as any of the following events at any time of the hospitalization: hypoxia requiring supplemental oxygen, need for mechanical ventilation, need for intensive care unit treatment, or death due to COVID-19. All patients underwent blood sample withdrawals and ECG recordings during active disease and recovery, specifically after that therapeutic interventions resulted in a significant clinical improvement along with a >60% decrease in IL-6 levels when compared to the baseline levels. In all cases, laboratory parameters were measured on the same day as the ECGs.

The QTc interval can be transiently but substantially influenced by a wide range of acquired factors, primarily electrolyte imbalances and medications. To better dissect the specific role of IL-6 in promoting QTc prolongation in COVID-19, strict inclusion criteria for patient enrollment were used in an effort to minimize the impact of confounders. Specifically, patients presenting with or developing electrolyte alterations (hypokalemia, hypocalcemia, and hypomagnesemia) were excluded. Patients taking QT-prolonging medications with conditional, possible, or known risk of torsades de pointes^1^ were excluded only if they were treated with the drugs during active disease but not at recovery, that is, those withdrawing liable drugs between these two time points (but keeping patients maintaining the same liable drug throughout). Moreover, due to the need for more complicated methods for QT interval correction, patients presenting with or developing atrial fibrillation/flutter were also excluded. The demographic and clinical features of patients with COVID-19 are presented in Table 1.

**TABLE 1:** Demographic, clinical, and laboratory characteristics of patients with COVID-19.

Patients, n	33
Age, years	63 (22.5)
Females, n	12/33 (36%)
**Clinical features**	
Dyspnea	14/33 (42%)
Respiratory failure	13/33 (39%)
Fever	11/33 (33%)
Gastrointestinal	4/33 (12%)
Cough, sneeze	4/33 (12%)
Asthenia	3/33 (9%)
Osteoarticular symptoms	2/33 (6%)
Lipothymia	1/33 (3%)
**Severe COVID-19**, n	33/33 (100%)
Respiratory support	33/33 (100%)
ICU admission	2/33 (6%)
Death	2/33 (6%)
**Respiratory support, n**	33/33 (100%)
*Oxygen therapy*	33/33 (100%)
Nasal cannulas	33/33 (100%)
VentiMask	29/33 (88%)
HFNC	10/33 (30%)
*Mechanic support*	19/33 (58%)
CPAP	19/19 (100%)
OTI	1/19 (5%)
Concomitant diseases*, n	6/33 (18%)
*Cardiac diseases*	5/6 (83%)
Acute coronary syndrome	3/5 (60%)
Left ventricular hypertrophy	1/5 (20%)
Chronic coronary artery disease	1/5 (20%)
*Extra-cardiac diseases*	1/6 (17%)
Diabetes mellitus type II	1/1 (100%)
Chronic kidney disease	1/1 (100%)
**QTc prolonging-drugs, n**	24/33 (73%)
Pantoprazole	20/24 (83%)
Indapamide	3/24 (13%)
Paroxetine	3/24 (13%)
Furosemide	2/24 (8%)
Dextetomedine	1/24 (4%)
Lansoprazole	1/24 (4%)
Esomeprazole	1/24 (4%)
Tacrolimus	1/24 (4%)
Venlafaxine	1/24 (4%)
Amiodarone	1/24 (4%)
Mean QT-drugs number per patient	1.0 ± 0.0.8
Mean QTc-prolonging risk factor number per patient**	1.3 ± 1.0
Patients without QT-prolonging risk factors, n	8/33 (24%)

*Diseases recognized to be a risk factor for QTc prolongation (14–20); **including diseases and QTc-prolonging drugs.

*COVID-19, Coronavirus disease 2019; HFNC, High flow nasal cannula; CPAP, Continuous positive airway pressure; OTI, Oreotracheal intubation.*

*Values are expressed as median (interquartile range), frequency count and percentages, or mean (±standard deviation).*

In addition, 20 healthy controls, comparable with COVID-19 patients in terms of age and sex, were included as a confirmatory group to further compare the ECG parameters of the COVID-19 cohort between patients with active disease and after recovery (Supplementary Table I).

### Electrocardiogram Recordings

In patients with COVID-19 and controls, heart rate (HR), RR, QT, and QTc (Bazett’s formula, QT/RR^1/2^) intervals were manually measured on a standard 12-lead ECG. QTc was considered to be prolonged if the interval was >450 ms in men or >470 ms in women, in accordance with the European regulatory guidelines (21, 22).

Detailed information on QTc measurements is provided in the online Supplementary Material.

### Laboratory Analysis

The methods followed for the measurement of CRP, IL-6, and other analytes in humans are detailed in the online Supplementary Material.

### Determination of Interleukin-6 Doses and Concentrations for the *in vivo* and *in vitro* Experiments

For the determination of IL-6 doses and concentrations employed in the *in vivo* and *in vitro* experiments, many factors were taken into account. First, we considered that, when compared to the patients with COVID-19, animals/cells would have been exposed to IL-6 for a significantly shorter period of time (days vs. minutes). Moreover, in patients with active COVID-19, it is expected that high circulating levels of the soluble form of the IL-6 receptor (sIL-6R) are present, which markedly enhances the biological effects of IL-6 on cardiomyocytes *via* the trans-signaling pathway (23). Since large amounts of sIL-6R are systemically released by shedding from hepatocytes and leukocytes several hours after an inflammatory process is activated (23), it was anticipated that sIL-6R was absent in cultured myocytes and that it is present at low levels only in guinea pigs either at baseline or 40 min after IL-6 administration (a time too short to elicit the above-mentioned inflammation-induced changes). Thus, we estimated that significantly higher IL-6 concentrations were needed in our experimental models to reproduce biological effects comparable to those observed *in vivo* in patients with COVID-19.

Based on these considerations, as well as on a previous work demonstrating that in a medium supplemented with elevated sIL-6R concentrations (25 μg/L), co-incubation with at least 20 μg/L (20 ng/ml) of IL-6 for 40 min was necessary to obtain evident electrophysiological effects in ventricular myocytes (marked effects with 80 μg/L) (19), we decided to employ in our experimental studies a 10-time higher concentration of IL-6, that is, 200 μg/L, in an effort to fill the gaps in biological response observed in patients with COVID-19.

Then, considering that human extracellular fluid is ∼20% of body weight, a concentration of 200 μg/L was matched to a human dose of IL-6 at 40 μg/kg (based on intravenous injection which was rapidly administered in the absence of any metabolites). In accordance with a 4.6 times dose conversion factor between humans and guinea pigs based on body surface area (25), IL-6 at 184 μg/kg was thereby used to conduct *in vivo* experiments in guinea pigs.

### *In vivo* Study in Guinea Pig

Electrocardiogram recordings before and after IL-6 (184 μg/kg) administration were performed in five adult guinea pigs, as described in the online Supplementary Material.

### Action Potential and I_Kr_ Current Recordings of Guinea Pig Left Ventricular Myocytes

The procedures followed for electrophysiology recording and enzymatic dissociation of single ventricular myocytes are detailed in the online Supplementary Material.

### Statistical Analysis

A sample size of 33 patients was estimated considering the two-sided Wilcoxon test, with alpha = 0.05, 1-beta = 0.80, and effect size = 0.5. A group of 20 controls was selected based on a two-sided Mann–Whitney test, with alpha = 0.05, 1-beta = 0.80, effect size = 0.85, and a 2:1 ratio between case and control.

Parametric or non-parametric analyses were carried out based on the Kolmogorov–Smirnov test. To compare active/recovery conditions, the paired *t*-test or the Wilcoxon test was used; comparisons between patients and controls were performed with the unpaired *t*-test or the Mann–Whitney test. Fisher’s exact test was used for qualitative variables. Correlation analyses were performed with Spearman’s rank test. The *p*-values ≤ 0.05 were considered to be significant (GraphPad InStat).

More details are provided in the online Supplementary Material.

## Results

### Characteristics of Patients With COVID-19

Thirty-three patients (36% women; median 63 years) hospitalized for severe COVID-19 and fulfilling the inclusion criteria were consecutively enrolled during the third wave of the COVID-19 outbreak from April to May 2021. On admission, the most common symptoms were dyspnea (42%), respiratory failure (33%), and fever (33%). All patients required respiratory support during hospitalization, including mechanical ventilation in 58% of the cases. Two subjects needed ICU treatment, and two died due to COVID-19-associated complications (bacterial superinfections) (Table 1).

Although the electrolyte levels were in the normal range in all the cases, most patients (∼75%) presented with other concomitant QT-prolonging risk factors of acquired origin, which primarily included medications and cardiac diseases. However, the global burden of QT-prolonging risk factors did not significantly change throughout the study period and was in absolute modest, that is, on average ∼1 per patient. Nine patients did not receive QT-prolonging medications, and eight were free of any QT-prolonging risk factor (Tables 1, 2).

**TABLE 2:** Changes in laboratory and electrocardiographic parameters in patients with COVID-19 (*n* = 33), during active disease and after therapeutic interventions resulting in a >60% decrease in IL-6 level when compared to the baseline.

	Active	Recovery	*p*
CRP, mg/dl (r.v. < 0.5)	7.4 (9.8)	0.6 (1.1)	**<0.001**
IL-6, pg/ml (r.v. < 7.1 pg/ml)	28.2 (31.2)	2.7 (3.4)	**<0.001**
QT,ms	400 (65)	413 (33.5)	0.13
RR,ms	800 (181)	968 (173)	**<0.001**
QRS,ms	94 (16)	97 (17.5)	0.13
Patients with bundle branch block, n	1 (3%)	1 (3%)	1.0
Heart rate, bpm	75 (18.5)	62 (11.5)	<**0.001**
QTc, ms	441 (39)	417 (32)	**<0.001**
Patients with prolonged QTc*, n	9 (26%)	2 (6%)	**0.027**
Patients with QTc > 500 ms, n	2 (6%)	0 (0%)	0.49
QTc-Fridericia, ms	426 (34)	411 (30)	**0.010**
QTc-Framingham, ms	427 (33)	412 (29)	**0.012**
Mean QT-drugs number per patient	1.0 ± 0.8	1.6 ± 0.9	0.31
Potassium, mEq/L (r.v.3.5–5.5)	4.1 (0.8)	4.3 (0.7)	0.22
Calcium, mg/dl (r.v.8–11)	9.1 (8.8)	9.3 (9.)	0.23
Magnesium, mg/dl (r.v.1.5–2.5)	2.1 (0.4)	2.2 (0.4)	0.96
Troponin, ng/ml (r.v. < 15)	10.5 (13)	8.0 (13.9)	0.08
Patients with increased troponin, n	11 (33%)	9 (27%)	0.79
BNP, pg/ml (r.v. < 500)	176.8 (493.0)	153.0 (53.0)	0.50
Patients with increased BNP, n	8 (24%)	7 (21%)	1.0
Creatinine, mg/dl (r.v.0.7–1.2)	0.77 (0.31)	0.76 (0.21)	0.10
paO_2_, mmHg (r.v.70–100)	78.0 (43.4)	93.6 (47.4)	0.33
paCO_2_ mmHg (r.v. 35–45)	35.0 (5.2)	35.3 (7.0)	0.80
pH (r.v.7.35–7.45)	7.46 (0.10)	7.45 (0.00)	0.83
P/F (r.v. > 4.0)	1.9 (1.4)	2.4 (2.0)	**0.009**

*CRP, C-reactive protein; IL-6, interleukin-6; QT, QT interval; RR, RR interval; QTc, heart rate-corrected QT interval based on the Bazett’s formula; QTc-Fridericia, heart rate-corrected QT interval based on the Fridericia’s formula; QTc-Framingham, heart rate-corrected QT interval based on the Framingham’s formula; BNP, brain natriuretic peptide; P/F, paO_2_/FiO_2_ ratio; r.v.: reference values.*

*Values are expressed as median (interquartile range) or mean ± standard deviation. Differences were evaluated by the two-tailed Student’s paired “t” test or the two-tailed Wilcoxon matched pairs test.*

*Men > 450 ms; Women > 470 ms.

### Heart Rate-Corrected QT Interval in Patients With Severe COVID-19 and Its Relationship With Inflammatory Markers

**FIGURE 1:**
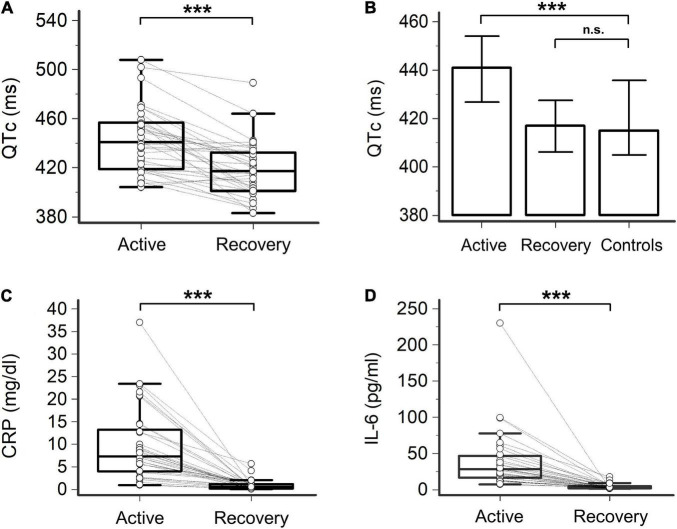
Heart rate-corrected QT interval (QTc), C-reactive protein (CRP), and interleukin-6 (IL-6) in patients with COVID-19, during active disease and recovery. **(A)** Heart rate-corrected QT interval based on Bazett’s formula (QTc); two-tail paired *t*-test, ***p < 0.001. **(B)** Comparison of QTc in patients with COVID-19, during active disease and recovery, and controls; two-tailed unpaired *t*-test, ***p < 0.001, n.s. not significant. **(C)** CRP; two-tailed paired *t*-test, ***p < 0.0001. **(D)** IL-6; two-tailed Wilcoxon matched pairs test, ***p < 0.001. Patients, *n* = 33; controls, *n* = 20.

When compared to controls, patients with severe COVID-19 infection during active disease showed increased median QTc duration (441 vs. 415 ms, *p* < 0.001) and a higher prevalence of QTc prolongation (26% vs. 0%, two-sided Fisher’s exact test, *p* = 0.0097). Marked QTc prolongation (>500 ms) was found in 6% of the cases (Figure 1, Table 2, and Supplementary Table I). Given that in patients with active COVID-19, HR was higher than in controls due to inflammation-induced sympathetic activation (17) (75 vs. 64 bpm; *p* = 0.0095, two-tailed unpaired *t*-test), QTc was also calculated with alternative formulas, to exclude that the above differences were biased by the known tendency of the Bazett’s formula to overestimate QTc at higher HRs. Also, in this case, a statistically significant difference between patients with COVID-19 and controls was demonstrated for median values of both QTc-Fridericia (426 vs. 409 ms; *p* = 0.0018, two-tailed unpaired *t*-test) and QTc-Framingham (427 vs. 409 ms; *p* = 0.0017, two-tailed unpaired *t*-test) (Table 2, Supplementary Figure I, and Supplementary Table I).

After enrollment, all the patients with COVID-19 received an anti-inflammatory treatment based on medium–high doses of glucocorticoids (dexamethasone or methylprednisolone; median starting daily dose: 8 and 80 mg, respectively). Three subjects were additionally treated with the antiviral drug remdesivir, and one with the Janus-kinase inhibitor baricitinib. None of the subjects received hydroxychloroquine or lopinavir/ritonavir, and only three received azithromycin, but only aimed at preventing bacterial superinfections. Treatment was associated with a marked decrease in both CRP (median reduction 92.6%) and IL-6 (88.9%) levels, which occurred in a relatively short period of time (median 7.0 days, mean 10.3 ± 7.6 days). As expected (IL-6 is recognized to be the key mediator responsible for hepatic CRP synthesis following inflammatory activation) (26), a strong correlation between CRP and IL-6 levels was observed over time (*r* = 0.77, *p* < 0.001, Spearman’s rank test). On the contrary, all the other laboratory parameters, including electrolyte levels, troponin, brain natriuretic peptide (BNP), blood gases, pH, and creatinine, showed median levels within normal ranges and did not significantly change throughout the study period (Table 2 and Supplementary Figure II).

**FIGURE 2:**
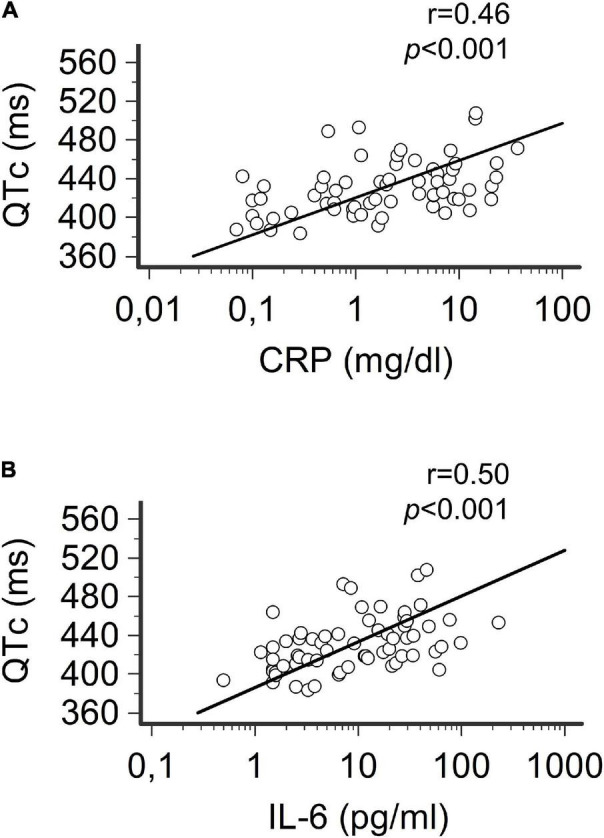
Correlation between QTc, CRP, and IL-6 in patients with COVID-19 over time. **(A)** Relationship between heart rate-corrected QT interval based on Bazett’s formula (QTc) and CRP levels. **(B)** Relationship between QTc and Il-6 levels.Spearman’s rank correlation. Patients, *n* = 33.

In this recovery phase, despite the persistence of concomitant QT-prolonging risk factors, a significant reduction in QTc duration (median ΔQTc = –24 ms, from 441 to 417 ms, *p* < 0.001) and QTc prolongation prevalence (from 26 to 6%, *p* = 0.027; QTc > 500 ms: 0%) was concomitantly observed, reaching values comparable to controls (Figure 1, Table 2, and Supplementary Table I). The length of QTc significantly correlated with CRP levels over time (*r* = 0.46, *p* < 0.001) and even more strongly with IL-6 levels (*r* = 0.50, *p* < 0.001) (Figure 2). These findings did not substantially change when the QT interval was corrected with alternative formulas. In fact, a significant, although less marked, decrease in QTc was also observed by using the Fridericia’s (ΔQTc = –15 ms, *p* = 0.010) or the Framingham’s (ΔQTc = –15 ms, *p* = 0.012) formulas, and again the values overlapped with those of controls (Table 2, Supplementary Figure I, and Supplementary Table I). Moreover, a significant correlation between QTc-Fridericia and QTc-Framingham duration and circulating IL-6 was also demonstrated in this case (Supplementary Figure III).

Although patients’ inclusion criteria minimized the impact of confounders, some subjects showed signs of acute myocardial strain or injury (as indexed by abnormal BNP or troponin, respectively), despite no significant differences between active and recovery phases. Moreover, most patients (25/33,76%) were treated with QT-prolonging drugs or showed other non-pharmacological QT-prolonging risk factors, on average ∼1 per patient, although stably present in both active and recovery phases (Table 1). To exclude that such concomitant factors in some way biased the results, several sensitivity analyses were performed by separately evaluating patients with and without myocardial involvement or concomitant QT-prolonging risk factors. In all cases, likewise in the whole population, QTc significantly decreased when the active and recovery phases were compared (Supplementary Figures IV, V), still showing a consistent correlation with IL-6 levels over time (Supplementary Table II). Moreover, QTc duration in the active phase always remained significantly increased in COVID-19 patients with respect to controls, even in the absence of abnormal troponin/BNP levels or concomitant QT-prolonging risk factors (Supplementary Figure VI). Nevertheless, in patients with signs of myocardial strain or injury, median QTc tended to be higher than in those without injury, a difference reaching the statistical significance when subjects with or without abnormal troponin levels were compared during the active phase (Supplementary Figure IV).

### Acute Effect of Interleukin-6 on Guinea Pig Heart Rate-Corrected QT Interval *in vivo* and on Action Potential Duration and I_Kr_ in Guinea Pig Ventricular Myocytes *in vitro*

**FIGURE 3:**
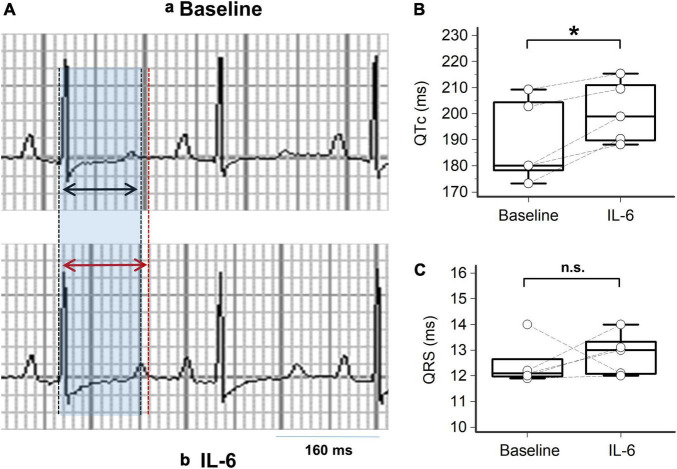
Impact of IL-6 on QTc in guinea pigs. **(A)** Representative lead-II ECG from a guinea pig during the basal condition **(a)** with a normal sinus rhythm at 250 beats/min with a QTc = 219 ms, and **(b)** at 40 min after intravenous injection with IL-6 (184 μg/kg) in the same guinea pig, demonstrating a sinus rhythm at 227 beats/min with a QTc = 232 ms; the shaded area in light blue and the black arrows indicate the QT interval at baseline, while the red arrows indicate the QT interval after IL-6. **(B)** Changes in QTc levels at baseline and 40 min after IL-6 injection for each guinea pig (*n* = 5); two-tailed paired *t*-test, *p < 0.05. **(C)** Changes in QRS interval observed at baseline and 40 min after IL-6 injection for each guinea pig (*n* = 5); two-tailed Wilcoxon matched pairs test, n.s. not significant.

Given that the above-mentioned clinical data point to a key role for IL-6 elevation in the pathogenesis of QTc prolongation associated with severe COVID-19, we conducted additional experiments in animal and cell models to further substantiate the individual significant impact of IL-6 in the phenomenon. First, IL-6 was directly administered intravenously into the guinea pigs (*n* = 5), a well-recognized animal model for QT interval studies because of the similarities between their ECG features and humans (27, 28). As presented in Figure 3 and Table 3, IL-6 administration was associated with a significant prolongation of QTc (mean ΔQTc: + 11.4 ms, *p* < 0.05), although no significant differences in the QRS duration were observed. Figure 3A shows representative ECGs of a guinea pig before and 40 min after IL-6 administration, respectively, with a ΔQTc of + 13 ms. Individual QTc and QRS data of guinea pigs are shown in Figures 3B,C, respectively.

**TABLE 3:** Changes in electrocardiographic parameters of guinea pigs (*n* = 5) before and after administration of interleukin-6 (IL-6).

	Baseline	IL-6	*p*
Heart rate, bpm	235.0 (13.0)	217.9 (12.9)	**0.0021**
RR, ms	258.4 (14.0)	279.4 (16.9)	**0.0059**
QRS, ms	12.4 (0.4)	12.8 (0.4)	0.62
QT, ms	120.0 (3.2)	130.6 (2.2)	**0.004**
QTc, ms	189.0 (7.1)	200.4 (5.3)	**0.014**

*Electrocardiogram was recorded at baseline and 40 min after intravenous injection with IL-6 (184 μg/kg).*

*Values are expressed as mean ± SEM.*

*Each guinea pig is used as its own control. Differences were evaluated by the two-tailed Student’s paired “t” test or the two-tailed Wilcoxon matched pairs test.*

*P values <0.05 are reported in bold.*

**FIGURE 4:**
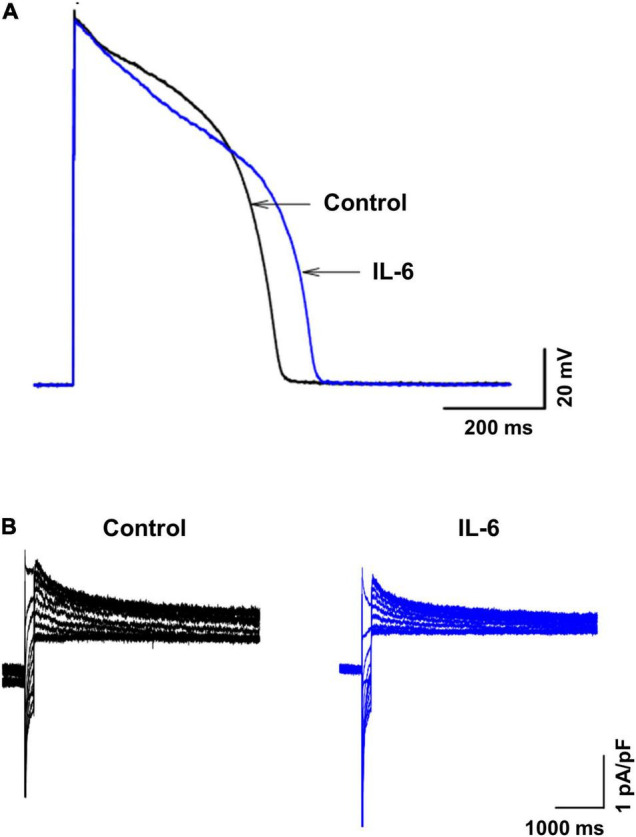
Effects of IL-6 on action potential and I_Kr_ in guinea pig ventricular myocytes. Representative action potentials **(A)** and I_Kr_ **(B)** recorded from a separate set of control myocytes (black traces) and from another set of myocytes preincubated with IL-6 (200 μg/L) for 40 min (blue traces). In panel **(A)**, action potentials were recorded in ventricular myocytes (*n* = 18) without IL-6 (black traces, control) and in ventricular myocytes (*n* = 11) preincubated with IL-6 (200 μg/L) for 40 min (blue traces) using the current-clamp mode. In panel **(B)**, I_Kr_ tail currents were recorded in ventricular myocytes (*n* = 10) without IL-6 (black traces) and in ventricular myocytes (*n* = 9) preincubated with IL-6 (200 μg/L) for 40 min (blue traces) using voltage-clamp mode.

Then, we investigated the mechanisms underlying the observed ECG changes by analyzing the *in vitro* effects of IL-6 on APD and I_Kr_ density of ventricular myocytes in guinea pigs. In fact, the APD is recognized to be a proxy of the QT interval and is critically affected by the I_Kr_ current. Incubation of the myocytes (*n* = 11) with IL-6 (200 μg/L) for 40 min resulted in a significant prolongation of the APD_90_ with respect to control cells (*n* = 18), in the absence of any appreciable change in the AP amplitude (Figure 4A and Table 4). Moreover, ventricular myocytes incubated with IL-6 (*n* = 9) showed a significant reduction in I_Kr_ density when compared to untreated cells (*n* = 10) (Figure 4B and Table 4).

**TABLE 4:** Effect of IL-6 on action potential and I_Kr_ of guinea pig left ventricular myocytes.

	APD_90_ (ms)	APA (mV)	I_Kr_ (pA/pF)
Control	430 ± 13.0 (*n* = 18)	136 ± 0.9 (*n* = 18)	0.79 ± 0.03 (*n* = 10)
IL-6	509 ± 13.0** (*n* = 11)	133 ± 1.0 (*n* = 11)	0.68 ± 0.03* (*n* = 9)

*APD_90_, action potential duration at 90%; APA, action potential amplitude; I_Kr_, rapid delayed rectifier current.*

*IL-6 was used at the concentration of 200 μg/L.*

*All values are expressed as mean ± SE. The number of experiments performed is indicated by “n = .”*

The unpaired t-test was used to compare data regarding IL-6 for AP, APA, and I_Kr_ with control. *P < 0.05, **P < 0.01 vs. control.

## Discussion

**FIGURE 5:**
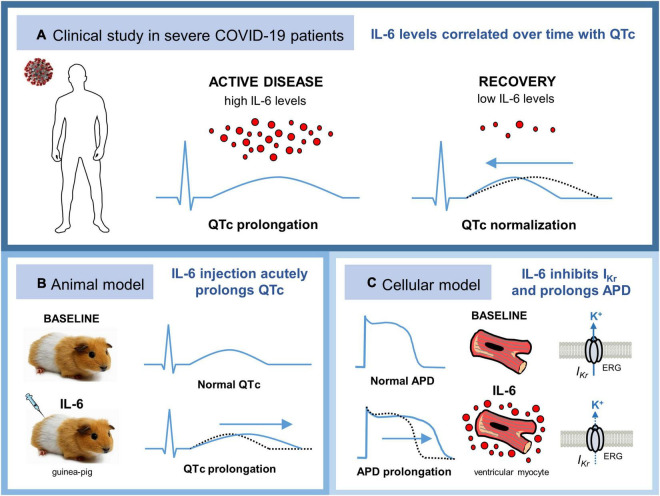
Systemic inflammatory activation may *per se* promote QTc prolongation in patients with severe COVID-19 *via* IL-6 elevation, leading to ventricular electric remodeling. **(A)** In patients with active severe COVID-19 and elevated IL-6 levels, QTc is significantly prolonged but rapidly normalized during recovery in correlation with IL-6 decrease. **(B)** Direct injection of IL-6 in an *in vivo* guinea pig model acutely prolongs QTc duration. **(C)** Guinea pig ventricular myocytes incubated *in vitro* with IL-6 show evident APD prolongation, along with significant inhibition in the I_Kr_ current. APD, action potential duration; COVID-19, coronavirus disease 2019; ERG, ether-a-go-go-related gene K^+^ channel; IL-6, interleukin-6; I_Kr_: rapid delayed rectifier potassium current; QTc, heart rate-corrected QT interval.

The key findings of the present study are the following: (1) in patients with active severe COVID-19 and elevated IL-6 levels, regardless of acute myocardial injury/strain and concomitant QT-prolonging risk factors, QTc is significantly prolonged and rapidly normalized in correlation with IL-6 decrease; (2) direct administration of IL-6 in an *in vivo* guinea pig model acutely prolongs QTc duration; and (3) ventricular myocytes incubated *in vitro* with IL-6 show evident APD prolongation, along with significant inhibition in the I_Kr_ current (Figure 5).

The substantial impact of systemic inflammation on ventricular repolarization is increasingly recognized (17). Many basic and translational studies clearly demonstrated that inflammatory cytokines directly affect cardiac electrophysiology, primarily by modulating the expression and functions of K^+^ channels (3, 17, 29–31). Moreover, accumulating clinical data indicate that during systemic inflammatory diseases, regardless of specific causes and pathogenesis, the QTc interval is frequently prolonged and associated with circulating levels of CRP and cytokines, particularly IL-6 (16, 18, 29). Based on such premises, it seems very likely that these mechanisms are also fully operative in patients with severe COVID-19, where high-grade systemic inflammation and elevated IL-6 levels are characteristically present. However, while many authors focused on the potential role of repurposed drugs (32) and cardiac strain/injury (6, 12) in the pathogenesis of COVID-19-associated QTc prolongation, no investigation was specifically designed to explore the discrete impact of IL-6 on this phenomenon. Thus, we herein analyzed the changes in QTc and IL-6 levels that occurred in patients with COVID-19 during the active disease and recovery phase, also making efforts to remove potential confounding effects due to concomitant QT-prolonging risk factors, pharmacological and non-pharmacological, and acute cardiac involvement.

In the present study, for the first time, we demonstrated the existence of a direct relationship between elevated IL-6 levels and QTc prolongation risk during the active phase of severe COVID-19 infection. In fact, in our cohort with high IL-6 levels (and substantially free from repurposed drugs), QTc duration and QTc prolongation prevalence in patients with COVID-19 were significantly increased when compared to controls. Notably, these values/frequencies were consistent with those found in large COVID-19 populations (4–6, 12), confirming that the cohort was well representative of the disease. In our COVID-19 patients, therapeutic interventions (mostly glucocorticoids) leading to IL-6 normalization were associated with a rapid (median 7 days) and a significant reduction in QTc length (∼25 ms) and QTc prolongation frequency (lower by over 4 times), until reaching values similar to those in controls. QTc correlated over time with CRP, a finding in agreement with a previous retrospective study by Gulletta et al. (20), and even more strongly with IL-6 levels. It is important to note that QTc changes occurred in the absence of significant modifications of electrolytes, troponin, or BNP, and without any variation in terms of concomitant QT-prolonging risk factors. Moreover, sensitivity analyses demonstrated that the significance of QTc modifications and their relationship with IL-6 also persisted when alternative QTc-correction formulas were used, or patient stratifications based on the presence/absence of abnormal BNP/troponin or concomitant QT-prolonging risk factors were performed. Nevertheless, patients with signs of acute myocardial involvement, particularly myocardial injury, showed median QTc values that tended to be higher when compared to those without. This finding, in agreement with the previous studies (6, 12) and supported by the experimental evidence that cardiac myocyte stretch and injury can lengthen APD, (33, 34) suggests that such electrophysiological changes in a cell emphasize the impact of IL-6 on ventricular repolarization.

The evidence that QTc modifications occurred in a few days, a time scale incompatible with cardiac structural modifications, points to direct, reversible effects of IL-6 on cardiac electrophysiology. Based on these findings, which are supportive of previous clinical data obtained in different inflammatory settings, (16, 18) several basic experiments were performed in the second “proof of concept” part of the study to demonstrate that IL-6 can exert relevant acute effects on ventricular repolarization, both *in vivo* and *in vitro*.

First, to discriminate the specific acute impact of this cytokine on surface ECG parameters in the absence of any potential confounding factor, an animal model was established by directly administering IL-6 to the guinea pigs. Forty minutes after administration, a significant increase in the QTc duration was demonstrated in these animals when compared to the baseline, despite no appreciable changes in ventricular depolarization duration. Interestingly, the relative magnitude of QTc prolongation was very similar to that observed in patients with COVID-19 (ΔQTc: 11.4 ms, 6.0% vs. 24.0 ms, 6.3%). Prompted by these data, which for the first time demonstrated that IL-6 is *per se* able to induce a measurable QTc lengthening in an animal model *in vivo*, we then conducted electrophysiological studies on ventricular myocytes from guinea pigs to provide insights into the underlying mechanisms. After incubation with IL-6 for 40 min, myocytes showed evident APD prolongation (∼80 ms, + 18%), along with a comparable decrease in the percentage of I_Kr_ density (–14%). These latter findings are consistent with two recent studies, one conducted by our group, in which significant APD prolonging effects were demonstrated for IL-6 in both guinea pig ventricular myocytes and in humans induced with pluripotent stem cell-derived cardiomyocytes (19, 35). These changes were associated with a significant I_Kr_ depression, without any appreciable effect on other currents (I_Na_ and I_K1_) (19). Overall, such basic data strongly indicate that IL-6 can prolong QTc duration *via* a direct and selective interference with ventricular repolarization, at least in part mediated by an inhibition of the I_Kr_ current.

One strength of our work is its translational approach, by which data obtained from the clinical study guided the following basic, proof of concept, in-vivo and in-vitro experiments. In fact, the hypothesis that IL-6 plays a key role in the pathogenesis of COVID-19-associated QTc prolongation, as suggested by the evidence that in these patients a correlation between IL-6 levels and QTc exists, was then supported by the demonstration that IL-6 can *per se* reproduce such ECG abnormality in an animal model, along with consistent electrophysiological changes in a cell.

Another strong point is the design of the study. This is the first prospective analysis of QTc in patients with COVID-19 where each patient served as their own control in the acute phase and recovery phase, simultaneously maintaining other concomitant QT-prolonging risk factors stable over the time period. In this regard, the treatment of COVID-19 patients with repurposed drugs, a potentially important confounder, was substantially absent in our cohort.

On the other hand, the fact that we conducted basic experiments using an IL-6 dose/concentration significantly higher than that found in patients with COVID-19 could represent a limitation. However, it should be considered that, when compared to animals/cells, patients were exposed to IL-6 for a significantly longer period of time (days vs. minutes), also in the presence of high circulating levels of the soluble form of the IL-6 receptor (systemically released by shedding from hepatocytes and leukocytes several hours after an inflammatory process is activated) (23), thus markedly enhancing the biological effects of IL-6 on cardiomyocytes *via* the trans-signaling pathway (19). In addition, measurement of IL-6 levels in humans quantifies only the free, circulating fraction of this cytokine, while no information is available on the fraction that is bound to receptors, which is actually responsible for the biological effects. Thus, the apparently high IL-6 concentrations used in our basic models are appropriate because they can more faithfully reproduce the biological conditions operating *in vivo* in patients with COVID-19.

Moreover, we have not tested the effects of IL-6 on other K^+^ currents, such as I_ks_, and this may represent a limitation of the study.

Finally, although many sensitivity analyses were performed to strengthen the validity of our data, the size of the clinical database is still a limitation and calls for larger studies.

## Conclusion

In conclusion, our data strongly suggest that during severe COVID-19 infection, systemic inflammatory activation can *per se* promote QTc prolongation *via* IL-6 elevation, leading to ventricular electric remodeling. Despite being transient, these modifications may have an important contributing role in explaining the higher propensity to arrhythmic events and associated poor outcomes in patients with COVID-19 (9, 10). Thus, a prompt and vigorous anti-inflammatory treatment based on glucocorticoids (as done in our study) and/or IL-6-targeted therapies (tocilizumab and sarilumab) could be a fundamental intervention not only to control the respiratory involvement, but also to reduce the arrhythmic risk, which can even be life-threatening if proper management is not provided. Although additional investigations are warranted, several lines of evidence support this hypothesis (40, 41). In particular, it has been demonstrated that tocilizumab can rapidly shorten QTc in rheumatoid arthritis, in parallel with inflammation and cytokine lowering agents (36, 37). Moreover, a large meta-analyses of randomized controlled trials recently demonstrated that glucocorticoid and/or IL-6 receptor antagonist therapy significantly reduced short-term mortality in patients with severe COVID-19 infection, including cardiovascular death (38, 39).

## Data Availability Statement

The original contributions presented in the study are included in the article/Supplementary Material, further inquiries can be directed to the corresponding author.

## Ethics Statement

The studies involving human participants were reviewed and approved by the Comitato Etico Regionale per la Sperimentazione Clinica della Regione Toscana, Sezione Area Vasta Sud Est. The patients/participants provided their written informed consent to participate in this study. The animal study was reviewed and approved by the Animal Care and Use Committee of Nanjing University of Chinese Medicine (#202005A045).

## Author Contributions

PL: conception and design of the work and drafting the work. RA, W-HZ, DV, MRB, XX, VS, K-SC, FS, AD’e, SB, TM, and MC: substantial contributions to the acquisition of data for the work. PL, MA, AC, GC, LC, PC, and MB: substantial contributions to the analysis of data for the work. PL, MA, FL-P, LC, PC, and MB: substantial contributions to the interpretation of data for the work. MA, AC, FL-P, LC, PC, and MB: revising the draft of the work critically for important intellectual content. All authors contributed to the final approval of the version to be published, agreement to be accountable for all aspects of the work in ensuring that questions related to the accuracy or integrity of any part of the work are appropriately investigated and resolved, and contributed to the article and approved the submitted version.

## Conflict of Interest

PL received a grant from Roche Italia S.p.A. outside the submitted work in 2018. The remaining authors declare that the research was conducted in the absence of any commercial or financial relationships that could be construed as a potential conflict of interest.

## Publisher’s Note

All claims expressed in this article are solely those of the authors and do not necessarily represent those of their affiliated organizations, or those of the publisher, the editors and the reviewers. Any product that may be evaluated in this article, or claim that may be made by its manufacturer, is not guaranteed or endorsed by the publisher.

## Footnotes

1
www.crediblemeds.org

## Abbreviations

AP: action potential
APD: action potential duration
COVID-19: coronavirus disease 2019
BNP: brain natriuretic peptide
CRP: C-reactive protein
ECG: electrocardiogram
HR: heart rate
hERG: human ether-a-go-go-related gene K^+^-channel
ICU: intensive care unit
IL-6: interleukin-6
I_Kr_: rapid delayed rectifier potassium current
QTc: heart rate-corrected QT interval
SARS-CoV-2: severe acute respiratory syndrome coronavirus-2
VAs: ventricular arrhythmias.
