# Supplementary material for: Interleukin-6 Elevation Is a Key Pathogenic Factor Underlying COVID-19-Associated Heart Rate-Corrected QT Interval Prolongation
Source: Front Cardiovasc Med. 2022 May 19;9:893681. doi: 10.3389/fcvm.2022.893681 (PMC9161021; doi:10.3389/fcvm.2022.893681)
Supplement: Supplementary file 1 [file Data_Sheet_1.PDF]

SUPPLEMENTAL MATERIAL

SUPPLEMENTAL METHODS

ECG recordings. The QT interval was measured between the onset of the Q wave or the onset of the QRS complex to the end of the T wave, defined as the return to the T-P baseline. QT interval was manually evaluated on standard 12-lead ECGs (25 mm/s and 10 mV/cm) from a commercially available recording system (Cardioline ECT WS 2000, Remco Italia, Vignate-Milano, Italy). A single investigator (M.A., cardiologist) blinded to the clinical and laboratory findings of the subjects, measured the QT interval on 3 non-consecutive beats (mean value), preferably in lead II or V5. Specifically, QT interval measurement was performed in most cases in lead II (COVID-19 patients: 31/33, 94%; healthy controls: 20/20, 100%). In only 2 patients the lead V5 was used, because the QT interval was not clearly measurable in lead II. In all cases, once a lead was selected to evaluate the QT interval during the active phase in a subject, the same lead was then used to perform the same measurement in the recovery phase. QT interval was then corrected for heart rate (HR) by the Bazett's formula (dividing the QT interval by the square root of the R-R interval, i.e. $QT/RR^{1/2}$) to obtain the heart rate-corrected QT interval (QTc).

Because of Bazett's formula might over- or underestimate QTc at higher and lower HRs, respectively, we additionally calculated QTc using alternative correction formulas, i.e. Fridericia [$QT/RR^{1/3}$] and Framingham [QT + 154 (1 − 60 x HR)].[1]

Laboratory analysis.

Blood samples were centrifuged at 3000xg and serum samples were stored at -80°C. C-reactive protein (CRP), interleukin-6 (IL-6), potassium (K^{+}), calcium (Ca^{++}), magnesium (Mg^{++}), troponin, brain natriuretic peptide (BNP), and creatinine were measured by an electrochemoluminescence-based immunoassay (COBAS-8000 platform, Roche Diagnostics GmbH; Mannheim, Germany) and values were reported as mg/dl (CRP, reference values <0.5; Ca^{++}, r.v.8-11; Mg^{++}, r.v.1.5-2.5;

creatinine, r.v. 0.7-1.2), ng/ml (troponin, r.v. <15), pg/ml (IL-6, r.v.<7.1; BNP, r.v. <500), or mEq/L (K^+, r.v.3.5-5.5), respectively.

Guinea-pig *in-vivo* study

Guinea pigs and humans have different potassium current balance (in guinea pigs, I_{Ks} current is more important than I_{Kr}, whereas in human I_{Kr} is predominant). In this study, we used the guinea pig model for several reasons: (1) it is a suitable in vivo animal model for QT studies because of the similarities of the ECG features with those in humans allowing for a clear delineation of the QT interval as well as the other ECG parameters;[2,3] (2) the relatively small size of the animal not requiring large amounts of test-reagents, like IL-6; (3) the high homology between human hERG and guinea-pig ERG channel (95.5% overall, and almost 100% at the extracellular pore region [E-pore] between S5 and S6).[3]

Dunkin-Hartley guinea pigs (300-350 g, age 4-5 weeks; equal number of females and males) from the experimental animal center of Nanjing University of Chinese Medicine (Nanjing, China) were used in this study. All experiments involving animals were approved by the Animal-Care and Use-Committee of Nanjing University of Chinese Medicine (#202005A045), whose policies adhere to the USA National Institutes of Health Guide for the Care and Use of Laboratory Animals. The study was carried out in compliance with the ARRIVE guidelines.

In anesthetized guinea pigs (20% urethane, 5 mL/kg, intraperitoneally), ECG recording (lead II) was performed by placing the leads at the left forelimb as the positive electrode, the right forelimb as the negative electrode and the hindlimb as the reference electrode. The HR, QRS, QT and QTc intervals ($QTc=QT/RR^{1/3}$) were analyzed. Recombinant human IL-6 (Pepro TECH, USA; catalog number 200-06) was dissolved in saline and injected intravenously (184 µg/kg). ECG recordings (sampling rate 1kHz) were obtained at basal conditions and 40 minutes after intravenous IL-6 administration. ECGs were analyzed using LabChart Pro software (RM6240BD, Biosignal analysis

system, China). At the completion of experiments, the deeply anaesthetized animals were sacrificed by cervical dislocation.

Action potential recordings from single ventricular myocyte

Guinea pig ventricular myocytes were obtained by enzymatic dissociation. Whole-cell current clamp configuration was used to record action potential (AP) using an amplifier (Axopatch 200B, Axon Instruments) as previously described.[4] The composition of internal solution was (mM): KCl 135, EGTA 10, Glucose 5, HEPES 10, Na_2-ATP 3, Na-GTP 0.5, pH 7.3 adjusted with KOH. The external solution contained (mM): NaCl 117, KCl 5.7, $NaHCO_3$ 4.4, $MgCl_2$ 1.7, HEPES 20, Glucose 20, Taurine 20, $CaCl_2$ 1.8, pH 7.4 with adjusted with NaOH. All experiments were performed at room temperature (20-22 °C). APs were elicited by passing appropriate current pulses at 0.1 Hz through the recording electrode to allow for any potential early afterdepolarizations to occur as they are rate dependent. AP durations at 90% full repolarization (APD_{90}) and the amplitude were measured. AP was analyzed using Sigmaplot 12.5 (Sigmaplot, Northampton, MA, USA).

Recording of I_{Kr} current from guinea pig left ventricular myocytes

Whole-cell patch clamp technique was used to record the rapid delayed rectifier current (I_{Kr}) from guinea pig ventricular myocytes. For recording, the external solution contained (mM): NaCl 145, KCl4.5, $MgCl_2$ 1, $CaCl_2$ 1.8, Glucose 10, HEPES 10, pH 7.4 adjusted with NaOH. The internal solution contained (mM): KCl 140, $MgCl_2$ 1, EGTA 11, HEPES 10, $CaCl_2$ 1, MgATP5, K_2ATP5, pH 7.30 with KOH. Currents were recorded in the whole-cell configuration of the patch-clamp technique using an Axopatch-200B amplifier (Axon Instruments, Inc, CA, USA). I_{Kr} was recorded from a holding potential (HP) of -50 mV using a short 200 ms depolarizing pulses from -40 to +70 mV in a 10-mV increment before returning to -40 mV for the tail current recording. L-type Ca^{2+} current (I_{CaL}) was blocked by the addition of 5 μM nifedipine in the bath solution and the slow

delayed rectifier K current (I_{Ks}) was blocked with 100 μM chromanol. The I_{Kr} current at +70 mV depolarizing potential was used in our experiments to maximize I_{kr} tail current. Current density analysis, was performed using Sigmaplot 12.5 (Sigmaplot, Northampton, MA, USA).

Statistical analysis

Descriptive statistics is reported as frequency count and percentage, for qualitative data, and median and interquartile range or mean±standard deviation, for quantitative data.

The following parametric or non-parametric statistical analyses were respectively carried out: the two-tail Student's paired t-test, or the two-tail Wilcoxon matched-pairs test, or the two-tail Student's unpaired t-test to evaluate differences in quantitative variables between two groups of data paired (changes in QT, QTc, RR, HR, QTc-Fridericia, QTc-Framingham, CRP, IL-6, electrolytes, troponin, BNP, creatinine, blood gases, and pH in COVID-19 patients, active *vs* recovery: changes in QT, QTc, QRS, RR, HR in guinea pigs, before and after IL-6) or unpaired (comparisons of QTc, QTc-Fridericia, QTc-Framingham in COVID-19 patients *vs* controls; comparisons of QTc in COVID-19 patients based on the presence or not of abnormal troponin/BNP, or QT-prolonging risk factors; electrophysiological experiments in cardiac myocytes), respectively; the Spearman rank correlation to verify possible statistical association between quantitative variables (in all cases not normally distributed) in COVID-19 patients (QTc *vs* CRP and IL-6); the two-sided Fisher's exact test was performed to evaluate statistical association between categorical variables in COVID-19 patients and controls (QTc prolongation prevalence, active *vs* recovery and patients *vs* controls). The Bonferroni's correction for multiple tests was applied when COVID-19 patients during active disease and recovery were compared to controls.

Supplemental-Table I.

Demographic, laboratory and electrocardiographic characteristics of healthy controls.

.

Subjects, n	20
Age, years	66 (18)
Females, n	7 (35%)
CRP, mg/dl (r.v.<0.5)	0.1 (0.2)
IL-6, pg/ml (r.v.<7.1 pg/ml)	0.2 (1.8)
QT,ms	394 (32.3)
RR,ms	933 (221.8)
Heart rate, bpm	64 (16.7)
QTc, ms	415 (37.5)
Patients with prolonged QTc*, n	0 (0%)
QTc-Fridericia, ms	409 (15.4)
QTc-Framingham, ms	409 (14.6)

CRP: C-reactive protein; IL-6: interleukin-6; QT: QT interval; RR: RR interval; QTc: heart rate-corrected QT interval based on the Bazett's formula; QTc-Fridericia: heart rate-corrected QT interval based on the Fridericia's formula; QTc-Framingham: heart rate-corrected QT interval based on the Framingham's formula; r.v: reference values.

Values are expressed as median (interquartile range), or frequency count and percentages.

* Males>450 ms/females>470 ms.

Supplemental-Table II.

Correlations between QTc and IL-6 in the presence or not of signs of myocardial injury or strain, or concomitant QT-prolonging risk factors.

	**n**	**r**	***p***
**All patients**	33	0.50	**<0.001**
**TPN+**	14	0.52	**0.004**
**TPN-**	19	0.50	**0.001**
**BNP+**	11	0.60	**0.003**
**BNP-**	22	0.45	**0.002**
**QT-risk factors+**	25	0.48	**<0.001**
**QT-risk factors-**	8	0.62	**0.009**

QTc: heart rate-corrected QT interval based on the Bazett's formula; TPN+/TPN-: patients with or without abnormal troponin levels (>15 ng/ml); BNP+/BNP-: patients with or without abnormal brain natriuretic peptide levels (>500 pg/ml); QT-risk factors+/ QT-risk factors -: patients with or without at least 1 concomitant QT-prolonging risk factor (pharmacologic or nonpharmacologic).

Correlations were evaluated by the Spearman test.

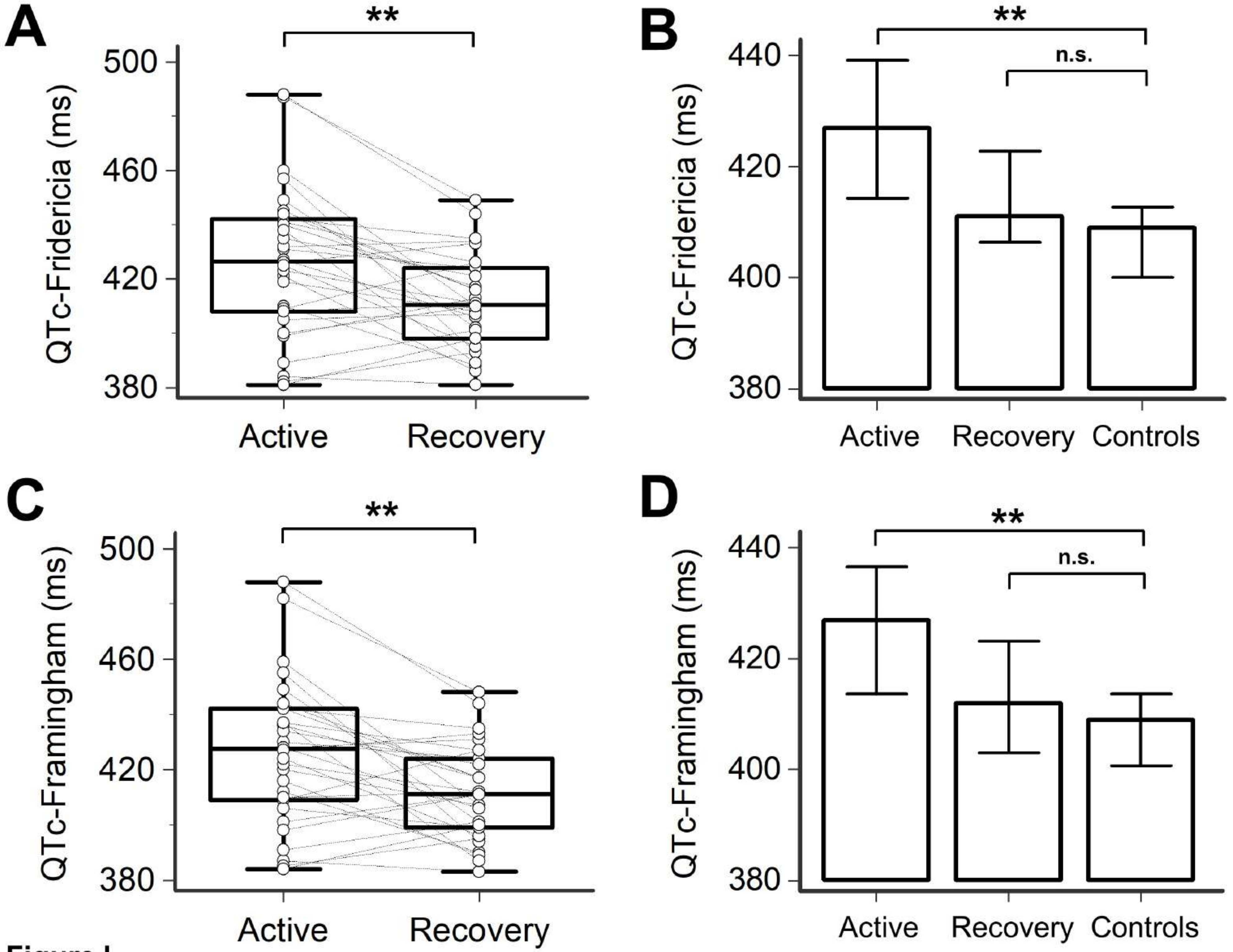
A
**
500
460
420
380
QTc-Fridericia (ms)
Active
Recovery
B
**
n.s.
440
420
400
380
QTc-Fridericia (ms)
Active
Recovery
Controls
C
**
500
460
420
380
QTc-Framingham (ms)
Active
Recovery
D
**
n.s.
440
420
400
380
QTc-Framingham (ms)
Active
Recovery
Controls

S-Figure I

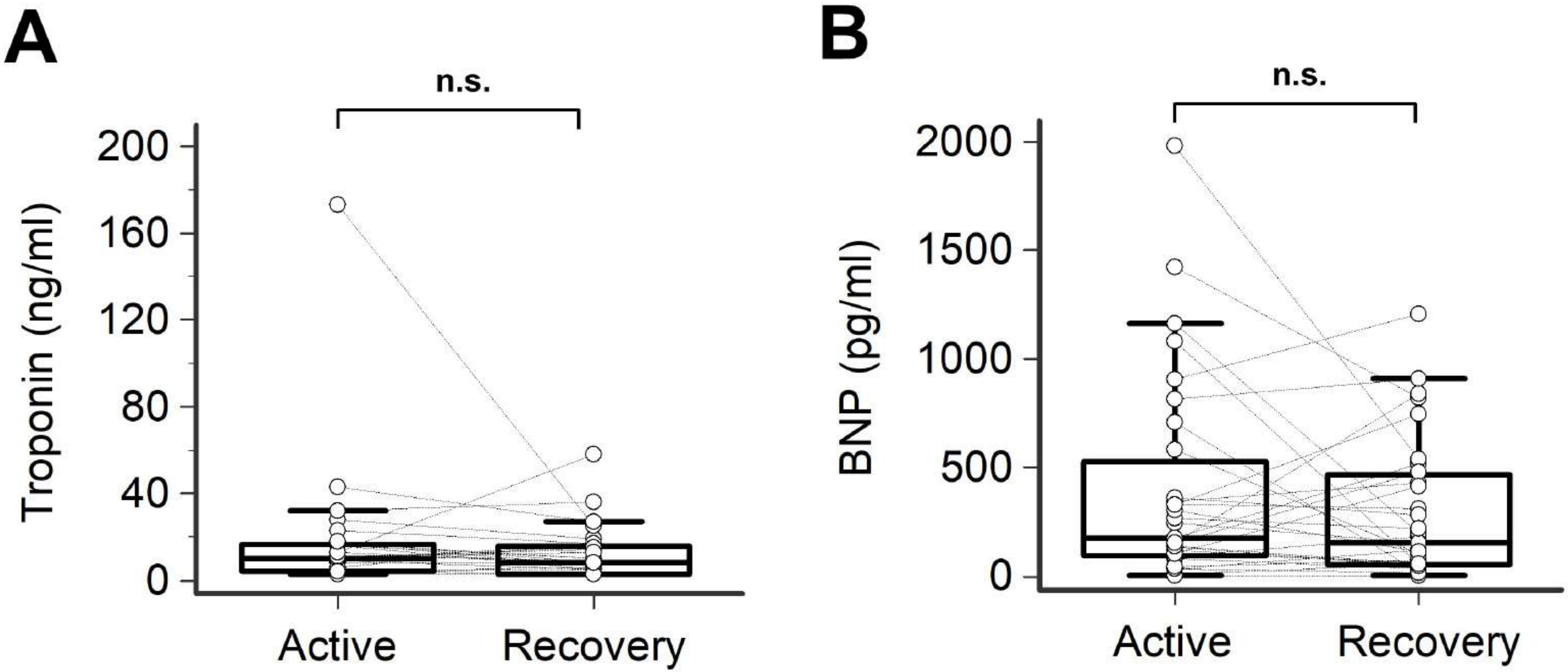
A
n.s.
200
160
120
80
40
0
Troponin (ng/ml)
Active
Recovery
B
n.s.
2000
1500
1000
500
0
BNP (pg/ml)
Active
Recovery

S-Figure II

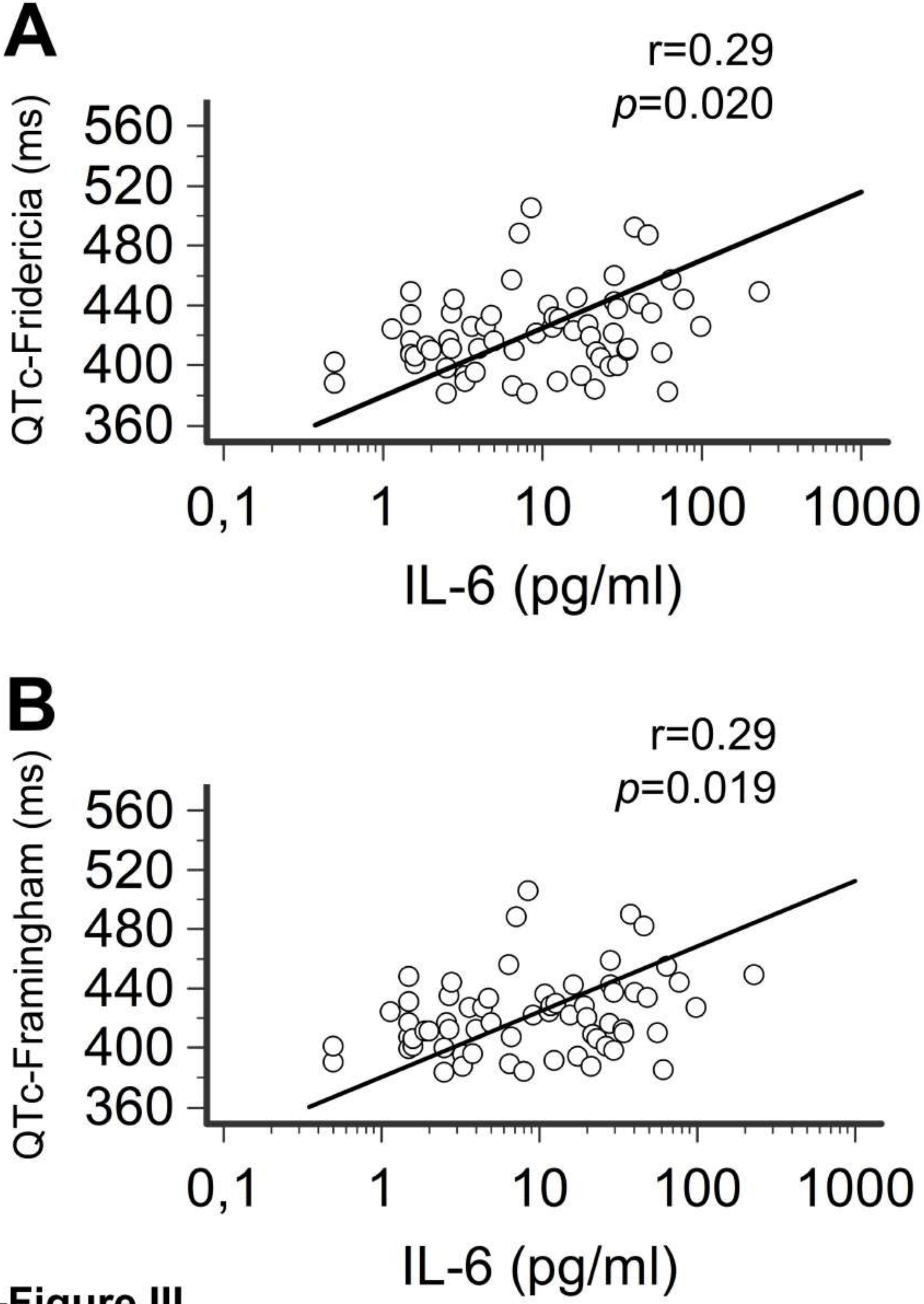
A
r=0.29
p=0.020
QTc-Fridericia (ms)
560
520
480
440
400
360
0,1
1
10
100
1000
IL-6 (pg/ml)
B
r=0.29
p=0.019
QTc-Framingham (ms)
560
520
480
440
400
360
0,1
1
10
100
1000
IL-6 (pg/ml)

S-Figure III

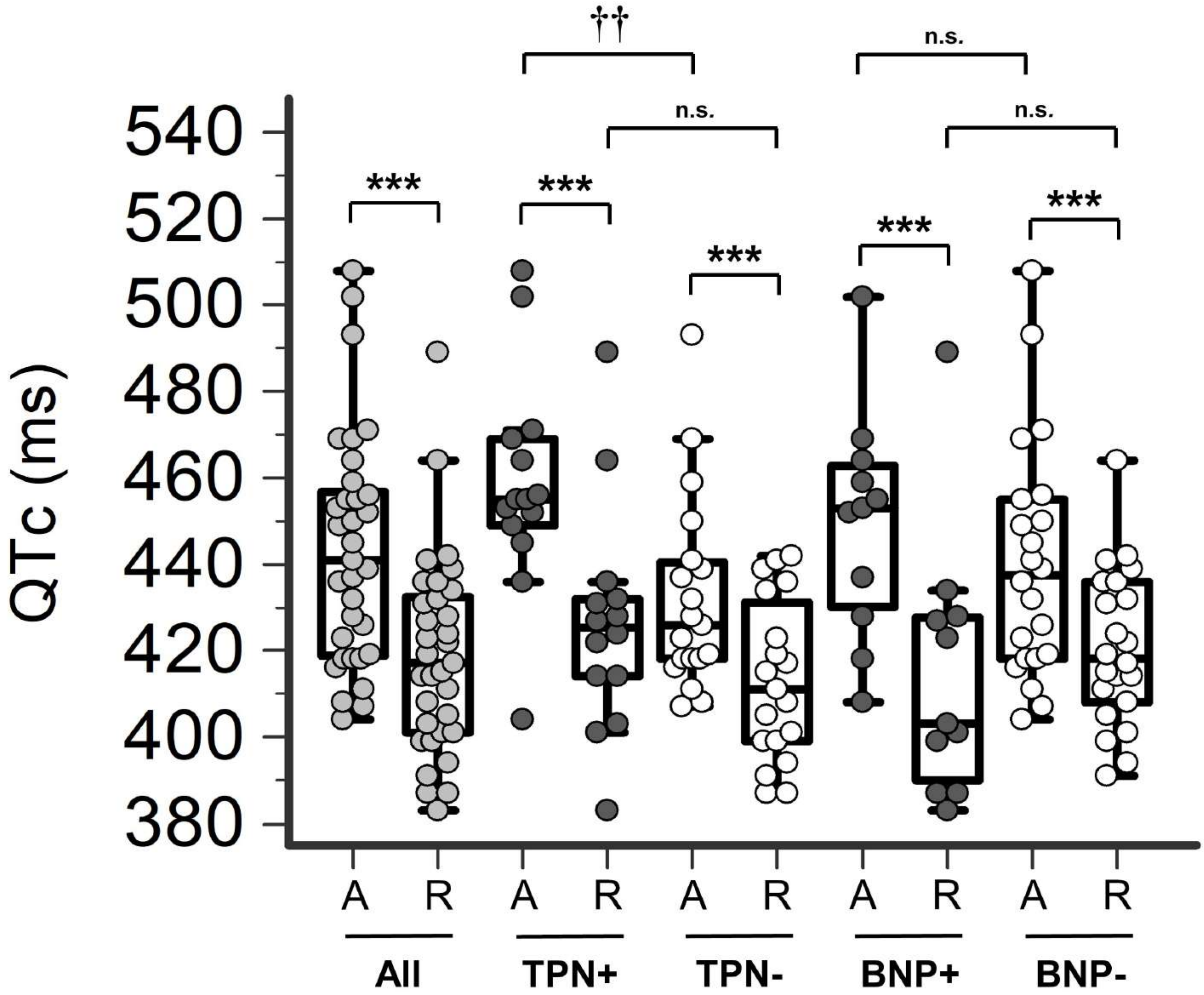

††
n.s.
n.s.
n.s.
***
***
***
***
***
QTc (ms)
540
520
500
480
460
440
420
400
380
A R A R A R A R A R
All
TPN+
TPN-
BNP+
BNP-

S-Figure IV

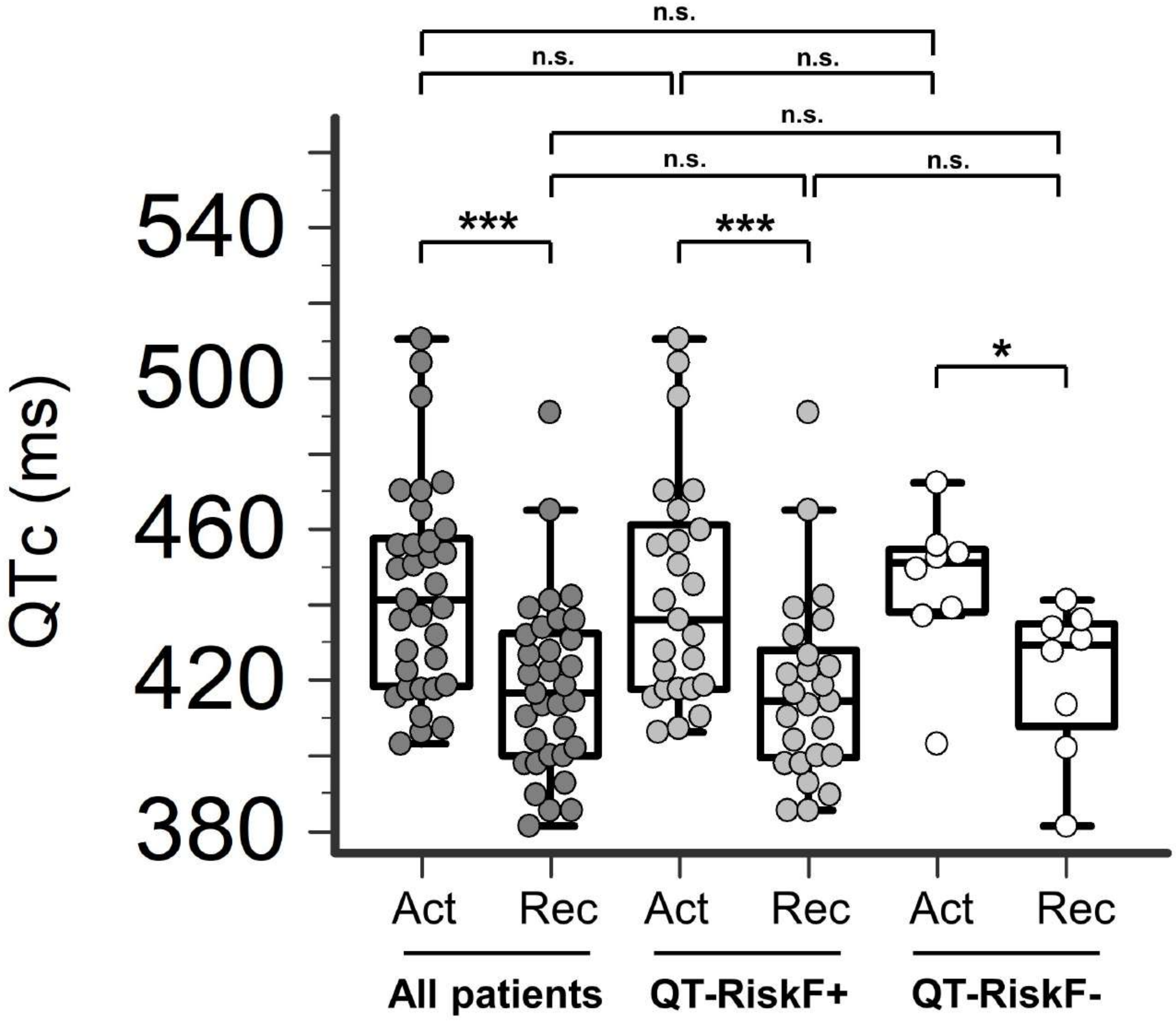
n.s.
n.s.
n.s.
n.s.
n.s.
n.s.
***
***
*
540
500
460
420
380
QTc (ms)
Act
Rec
Act
Rec
Act
Rec
All patients
QT-RiskF+
QT-RiskF-

S-Figure V

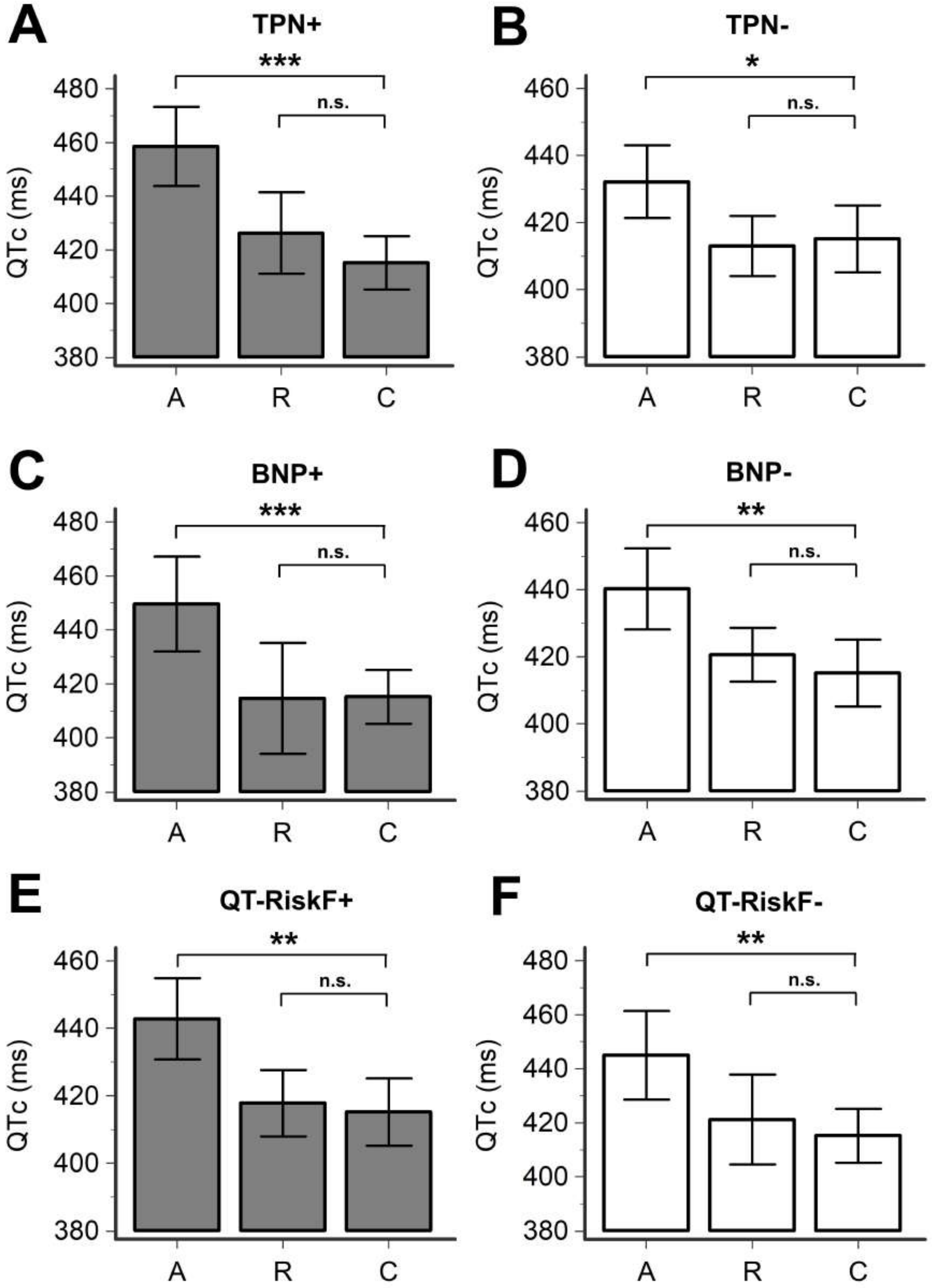

A
TPN+
***
n.s.
QTc (ms)
480
460
440
420
400
380
A
R
C
B
TPN-
*
n.s.
QTc (ms)
460
440
420
400
380
A
R
C
C
BNP+
***
n.s.
QTc (ms)
480
460
440
420
400
380
A
R
C
D
BNP-
**
n.s.
QTc (ms)
460
440
420
400
380
A
R
C
E
QT-RiskF+
**
n.s.
QTc (ms)
460
440
420
400
380
A
R
C
F
QT-RiskF-
**
n.s.
QTc (ms)
480
460
440
420
400
380
A
R
C

S-Figure VI

LEGEND TO SUPPLEMENTAL-FIGURES

S-Figure I. QTc-Fridericia and QTc-Framingham in COVID-19 patients, during active disease and recovery, and comparisons to controls. (**A**) Heart rate-corrected QT interval based on the Fridericia's formula (QTc-Fridericia); two-tail paired t-test, **p≤0.01. (**B**) Comparison of QTc-Fridericia in COVID-19 patients, during active disease and recovery, and controls; two-tail unpaired t-test, **p≤0.01, n.s. not significant. (**C**) Heart rate-corrected QT interval based on the Framingham's formula (QTc-Framingham); two-tail paired t-test, **p≤0.01. (**D**) Comparison of QTc-Framingham in COVID-19 patients, during active disease and recovery, and controls; two-tail unpaired t-test, **p≤0.01, n.s. not significant.

Patients, n=33; controls, n=20.

S-Figure II. Troponin and brain natriuretic peptide (BNP) levels in COVID-19 patients, during active disease and recovery. (**A**) Troponin; two-tail Wilcoxon matched-pairs test, n.s. not significant. (**B**) BNP; two-tail Wilcoxon matched-pairs test, n.s. not significant.

Patients, n=33.

S-Figure III. Correlation between QTc-Fridericia and QTc-Framingham with IL-6 in COVID-19 patients over time. (**A**) Relationship between heart rate-corrected QT interval based on the Fridericia's formula (QTc-Fridericia) and interleukin(IL)-6 levels. (**B**) Relationship between heart rate-corrected QT interval based on the Framingham's formula (QTc-Framingham) and IL-6 levels.

Spearman rank correlation. Patients, n=33.

S-Figure IV. QTc in COVID-19 patients, during active disease and recovery, based on the presence or absence of signs of myocardial injury or strain. Comparisons of heart rate-corrected QT interval based on the Bazett's formula (QTc) during active disease (A) and recovery (R) in the

whole COVID-19 population (Total, n=33), in patients with (TPN+, n=14) or without (TPN-, n=19) abormal troponin levels (>15 ng/ml), and in patients with (BNP+, n=11) or without (BNP-, n=22) abormal brain natriretic peptide (BNP) levels (>500 pg/ml). two-tail paired t-test (***p<0.001) or unpaired t-test (††p<0.01, n.s. not significant). *P*>0.05 for all comparisons between the whole population and subgroups in active and recovery phase, respectively (unpaired t-test).

S-Figure V. QTc in COVID-19 patients, during active disease and recovery, based on the presence or not of concomitant QT-prolonging risk factors (pharmacologic or nonpharmacologic). Comparisons of heart rate-corrected QT interval based on the Bazett's formula (QTc) during active disease (Act) and recovery (Rec) in the whole COVID-19 population (n=33), and in patients with (QT-RiskF+, n=25) or without (QT-RiskF-, n=8) concomitant QT-prolonging risk factors. Two-tails paired t-test (***p<0.001, *p<0.05) or unpaired t-test (n.s. not significant).

S-Figure VI. QTc in COVID-19 patients stratified by the presence or absence of abnormal troponin, brain natriuretic peptide (BNP) or QT-prolonging risk factors, during active disease and recovery, and comparisons to controls. (**A**) Comparison of heart rate-corrected QT interval based on the Bazett's formula (QTc) in COVID-19 patients with abnormal troponin levels (TPN+), during active disease (A) and recovery (R), and controls (C); two-tail unpaired t-test, ***p<0.001, n.s. not significant. (**B**) Comparison of QTc in COVID-19 patients without abnormal troponin levels (TPN-), during active disease and recovery, and controls; two-tail unpaired t-test, *p<0.025, n.s. not significant. (**C**) Comparison of QTc in COVID-19 patients with abnormal BNP levels (BNP+), during active disease and recovery, and controls; two-tail unpaired t-test, ***p<0.001, n.s. not significant. (**D**) Comparison of QTc in COVID-19 patients without abnormal BNP levels (BNP-), during active disease and recovery, and controls; two-tail unpaired t-test, **p<0.01, n.s. not significant. (**E**) Comparison of QTc in COVID-19 patients with QT-prolonging risk factors (QT-

RiskF+), during active disease and recovery, and controls; two-tail unpaired t-test, **p<0.01, n.s. not significant. (**F**) Comparison of QTc in COVID-19 patients without QT-prolonging risk factors (QT-RiskF-), during active disease and recovery, and controls; two-tail unpaired t-test, **p<0.01, n.s. not significant.

Patients, n=33; controls, n=20.

References:

1. Chiladakis J, Kalogeropoulos A, Arvanitis P, Koutsogiannis N, Zagli F, Alexopoulos D. Heart rate-dependence of QTc intervals assessed by different correction methods in patients with normal or prolonged repolarization. *Pacing Clin Electrophysiol.* 2010;33:553-60.

2. Hamlin RL, Kijtawornrat A, Keene BW, Hamlin DM. QT and RR intervals in conscious and anesthetized guinea pigs with highly varying RR intervals and given QTc-lengthening test articles. *Toxicol Sci*. 2003;76:437-42.

3. Yue Y, Castrichini M, Srivastava U, Fabris F, Shah K, Li Z, Qu Y, El-Sherif N, Zhou Z, January C, Hussain MM, Jiang XC, Sobie EA, Wahren-Herlenius M, Chahine M, Capecchi PL, Laghi-Pasini F, Lazzerini PE, Boutjdir M. Pathogenesis of the Novel Autoimmune-Associated Long-QT Syndrome. *Circulation*. 2015;132:230-40.

4. Zhang M, Xie M, Li S et al. Electrophysiologic Studies on the Risks and Potential Mechanism Underlying the Proarrhythmic Nature of Azithromycin. *Cardiovasc Toxicol* 2017;17:434-440.
